# Isolated Perforation of Multiple Jejunal Diverticulae: A Very Rare Cause of Acute Abdomen

**DOI:** 10.7759/cureus.52228

**Published:** 2024-01-13

**Authors:** Shariful Islam, Aneela Shah, Vijay Naraynsingh

**Affiliations:** 1 General Surgery/Oncoplastic Breast Surgery, San Fernando General Hospital, San Fernando, TTO; 2 Clinical Surgical Sciences, University of the West Indies, St. Augustine, TTO; 3 General Surgery, San Fernando General Hospital, San Fernando, TTO; 4 Surgery, Medical Associates Hospital, St. Joseph, TTO

**Keywords:** peritonitis, laparotomy, jejunum, emergency, bowel resection, diverticulitis, perforation, acute abdomen, abdominal pain, jejunal diverticulitis

## Abstract

Isolated jejunal diverticular (JD) perforation is extremely rare; it usually presents as a diagnostic dilemma and is often discovered incidentally on laparotomy. Most of these perforations are single. Literature has revealed only one case of multiple small bowel diverticular perforations. We report the first case of simultaneous perforation of four jejunal diverticulae in an 85-year-old male. Small bowel resection and primary anastomosis were performed. The patient had an uneventful post-operative recovery. This case highlights the importance of prompt diagnosis and timely management to reduce the morbidity and mortality of these patients. It should be included in the differential diagnosis in all elderly patients presenting with acute abdomen.

## Introduction

Jejunal diverticulosis is characterized by herniations of the mucosa and submucosa through the muscular layer of the bowel. These false diverticulae are thought to occur through weaknesses, at sites where the vasa recta enter the muscularis propria. This is a rare finding, with an estimated annual incidence of 0.06-2.3% [1-3]. It is rarer than colonic diverticulosis and occurs in the sixth and seventh decades with a male predominance. Its etiology is unknown and, while often asymptomatic, 10-30% of patients develop non-specific symptoms. A small number of patients present with life-threatening complications like diverticulitis, bleeding, obstruction, and perforation [2, 3]. Such complications must be promptly diagnosed to reduce the high risk of morbidity and mortality. We report a case of an elderly male who presented with an acute abdomen and a computerized tomography scan suggesting a perforated viscus. Surgical findings at laparotomy revealed multiple large perforated jejunal diverticulae. We describe the approach to the diagnosis and management of this phenomenon.

## Case presentation

An 85-year-old male with no comorbidities was brought to our emergency department with a one-week history of central abdominal pain, which has worsened over the last 24 hours. This was associated with decreased appetite but no other acute gastrointestinal symptoms. The patient also gave a history of constipation for the last two days. He had no history of smoking or alcohol consumption and was able to perform all activities of daily living. On examination, he appeared comfortable, mildly tachycardic with a pulse rate of 102 beats per minute but normotensive and SPO2 of 96% at room temperature. Abdominal examination revealed generalized tenderness with guarding and rebound tenderness and decreased bowel sounds. The digital rectal examination was normal.

**Figure 1:**
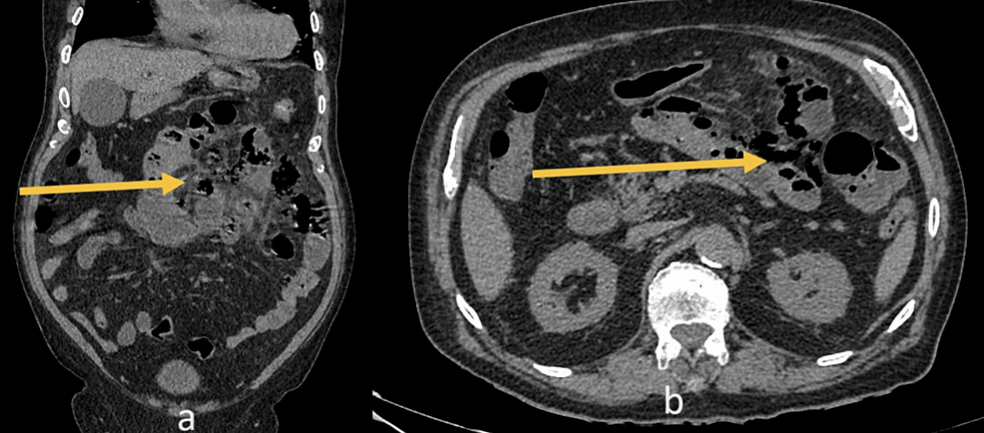
Contrast CT images of the abdomen and pelvis (a) coronal (b) cross-sectional views showing small bowel perforation with free air in the left upper quadrant (yellow arrows)

Blood investigations revealed an elevated white blood cell count of 15 x 10^9^/L, hemoglobin 12.6 gm/dl, and a normal renal function test. An arterial blood gas analysis revealed a moderate metabolic acidosis (i.e., pH - 7.29, PaCO2 - 26 mmHg, PaO2 - 92%, HCO2 - 18 mEq/L). An abdominal contrast computed tomography (CT) scan showed multiple locules of free air within the small bowel mesentery in the left upper quadrant, with mesenteric fat stranding (Figure 1). A diagnosis of a perforated viscus was made and the patient was resuscitated. A broad-spectrum intravenous antibiotic was started and the patient consented for emergency laparotomy. The patient was reviewed by the cardiologist as well as by the anaesthesiologist before the surgery.

**Figure 2:**
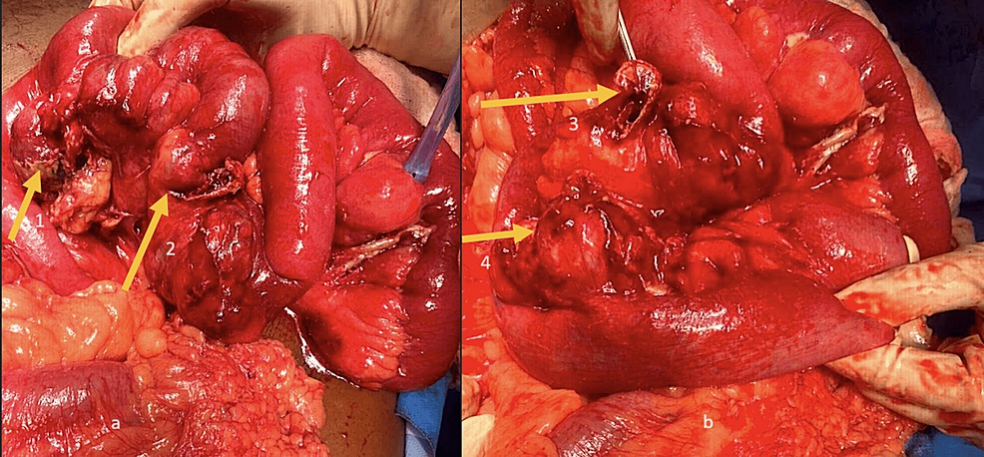
Surgical pictures (a) showing 1st & 2nd, (b) showing 3rd & 4th perforations of jejunal diverticulae (yellow arrows)

At surgery, multiple (four) large perforated diverticulae were identified along the proximal jejunum, with associated inter-loop abscesses (Figure 2). No other significant pathology was identified. Resection of the affected segment of the proximal jejunum was performed and a side-to-side stapled anastomosis was performed. The patient had an uneventful postoperative recovery and he was discharged home on day 5 after the surgery. The patient was followed up in the surgical outpatient clinic and during his last visit to the clinic at 3 months, he was doing well without any further complaints.

## Discussion

Diverticular disease is a relatively common disorder that commonly affects the colon. Small bowel diverticulosis, however, while rarer, is a well-recognized entity that may be incidentally found or present with acute life-threatening complications. Somerling and Baille first described small bowel diverticulosis in 1794 [1, 3]. It's unclear exactly what causes it, although some research has linked intestinal dyskinesia and aberrant neuromotor innervation to increased intraluminal pressure leading to the formation of pulsion diverticulae along the mesenteric border where the vasa recta penetrate the small intestinal wall [1].

This condition is more prevalent in the elderly with a peak incidence in the sixth and seventh decades of life [1, 4]. However, reports of it in children [5] and young adults [6] have also been made. It is predominately reported in males. In most cases, jejunal diverticulosis is diagnosed incidentally (either on imaging or intra-operatively). However, around 29% of patients present with symptoms including vague abdominal pain and early satiety, very rarely it can also present as an incarcerated hernia [7]. Management of asymptomatic or minimally symptomatic jejunal diverticulosis is similar to that of colonic diverticulosis [1]. Ten to thirty percent of patients develop significant complications like diverticulitis, perforation, bleeding, or small bowel obstruction. Perforation generally leads to peritonitis, sepsis, intra-abdominal abscesses, or fistulae [3, 5-7]. However, the symptoms are also not specific to target a diagnosis of jejunal diverticular perforation. Other causes of jejunal perforation like foreign bodies and trauma should also be considered in the differential [4].

Perforation, although rare (occurring in 2.1-7% of diverticulitis cases), poses a significant risk with high mortality rates [8, 9]. Cases of perforation typically present acutely with symptoms and signs of peritonitis, including fever and severe abdominal tenderness. The complications of diverticula can be treated through conservative or surgical management, especially in cases of perforation [8-10]. Isolated jejunal diverticular perforation is extremely rare (2-6% of acute diverticulitis) and is associated with mortality, especially in elderly patients [8, 9]. Most of these perforations are single and only a few cases have been reported in the English literature [3]. Documentation of multiple jejunal perforations in a single case appears to be even rarer [11].

Imaging plays an important role in establishing the causes of acute abdominal pain. Plain films of the abdomen can detect the presence of a pneumo-peritoneum [12], which generally leads to urgent laparotomy and identification of the pathology. CT may be more helpful in identifying clues regarding the actual site of visceral perforation [13]. In our case, CT localized the pneumo-peritoneum to the left upper quadrant, with inflammatory changes near the small bowel. The presence of jejunal diverticulae, however, was not identified. While these features narrowed our differential diagnosis in favor of small bowel perforation, it did not change the patient’s management with emergent laparotomy.

Perforated jejunal diverticulitis is managed based on the clinical presentation and fitness of the patient. Surgeons must also be wary of the mortality rate, which can be as high as 21% to 40%, especially in elderly patients, those with significant comorbidities, and those with severe sepsis [7, 14]. Conservative treatment is usually recommended in these groups of patients as surgery is often contraindicated [13, 15].

The standard method of treatment involves exploratory laparotomy and resection of the afflicted segments of the small bowel, with or without primary anastomosis [9]. However, resection should be limited only to the involved and unhealthy segment of the small intestine to avoid short bowel syndrome [3, 9, 16]. Literature has reported several other techniques, i.e., diverticulectomy, primary repair of the perforation, and omental patch closure; however, these should be avoided in cases of serious sepsis and contamination [4, 11, 17]. These techniques can also be challenging to execute as jejunal diverticulae may be relatively inaccessible as it is next to the mesentery. When compared with segmental bowel resection these techniques are associated with a higher mortality rate [15].

The laparoscopic approach may also be a good alternative in cases where it is safe and feasible. The use of the laparoscope can guide the diagnosis of perforated diverticulitis and can also aid in planning the best approach to treating the pathology. Additionally, a minimally invasive approach is well known to be associated with more satisfactory postoperative outcomes [15, 18]. Interestingly, non-operative management of jejunal diverticular perforations is a relatively new approach in patients who are not peritonitic with localized contamination. However, evidence is limited to individual case reports. Novak et al. described a case series treated conservatively with antibiotics and CT-guided drainage of abdominal abscesses [19].

## Conclusions

Small intestinal diverticulosis and its complications are important to consider in the differential diagnosis of an acute abdomen, especially in the elderly population. Perforated jejunal diverticulitis can be both a diagnostic and therapeutic challenge with a high mortality rate. There are no established guidelines for treatment due to the rarity of this pathology. The appropriate approach to management depends on the patient's presentation and stability. While most reports describe singular perforated diverticulae, this case report serves to highlight the rarity of multiple perforated diverticulae occurring in the same index case.
